# Multiplex Detection of Biogenic Amines for Meat Freshness Monitoring Using Nanoplasmonic Colorimetric Sensor Array

**DOI:** 10.3390/bios13080803

**Published:** 2023-08-10

**Authors:** Samira Abbasi-Moayed, Afsaneh Orouji, Mohammad Reza Hormozi-Nezhad

**Affiliations:** 1 Department of Analytical Chemistry, Faculty of chemistry, Kharazmi University, Tehran 15719-14911, Iran; 2 Department of Chemistry, Sharif University of Technology, Tehran 11155-9516, Iran; afiorouji@gmail.com; 3 Institute for Nanoscience and Nanotechnology, Sharif University of Technology, Tehran 11155-9516, Iran

**Keywords:** colorimetric, sensor array, biogenic amines, meat freshness, nanoplasmonic

## Abstract

Biogenic amines (BAs) were presented as significant markers for the evaluation of the spoilage of meat and meat products. In this work, a colorimetric sensor array was developed for the discrimination and detection of spermine (SP), spermidine (SD), histamine (HS), and tryptamine (TP) as important BAs in food assessment. For this aim, two important spherical plasmonic nanoparticles, namely gold nanoparticles (AuNPs) and silver nanoparticles (AgNPs), were utilized as the sensing elements of the probes. The cross-reactive interaction of the target biogenic amines and the plasmonic nanoparticles caused the aggregation-induced UV–Vis spectra changes, which were accompanied by visual color variation in the solution. The collected responses were analyzed by principal component analysis-linear discrimination analysis (PCA-LDA) to classify the four BAs. This colorimetric sensor array can also discriminate between the individual BAs and their mixture accurately. Partial least squares regression (PLS-R) was also utilized for quantitative analysis of the BAs. The wide linear concentration ranges of 0.1–10.0 µM for the four BAs and desirable figures of merits (FOMs) showed the potential of the developed sensor for quantitative detection of the BAs. Finally, the practical ability of the developed probe was studied by the determination of the BAs in the meat samples, which successfully proved the potential of the colorimetric sensor array in a food sample.

## 1. Introduction

The safety and quality control of food products are one of the most important concerns for food consumers and health organizations all around the world [1]. The World Health Organization (WHO) reported that more than two hundred diseases are related to the quality of food and a vast number of people have contracted a food illness during their life [1]. Assessment of food quality, especially quality loss in meat products, can be evaluated by physical characteristics such as changes in structure, texture, color, and leakage of water [2]. Despite the common advantages of considering the physical characteristic, the enormous heterogeneity in the amount of connective tissues, fat contents, and the presence of bones limited the application of physical measuring methods [3,4]. Therefore, chemical analysis can be introduced as an important and complementary method that can be used when it is required to evaluate the freshness of meat products quickly and unambiguously [3]. Biogenic amines (BA) have been introduced as a toxic target by the Food and Drug Administration (FDA) [5,6]. Generally, BAs, which are nitrogen-containing organic bases, can be used as chemical biomarkers for the evaluation of food quality, especially meat products [7,8]. Therefore, monitoring the concentration of BAs during food production helps to control the quality and freshness [1]. The concentration of BAs is very low in fresh meat and meat products. However, the contents of BAs are increased during the amino acid decarboxylation and spoilage of meat products [5,9]. There are a number of BAs, including spermine (SP), spermidine (SD), tryptamine (TP), and histamine (HS), that are related to meat freshness [1,10]. These BAs play critical biological roles in the body, such as their essential role in growth, renewal, and metabolism in all organs of the body, temperature regulation, nutrition intake, and alternation of blood pressure [11,12]. Despite their beneficial roles, the ingestion of high levels of BAs can cause serious health diseases with specific symptoms such as nausea, hyper and hypotension, renal intoxication, heart tremor, headaches, cerebral hemorrhage, and even death [13,14]. Therefore, to prevent food poisoning, developing a highly sensitive and reliable sensor for the determination of these food safety indicators is of great importance.

A vast number of studies have been presented for the detection of BAs in different types of food samples [7]. Most of these techniques, including gas chromatography-mass spectrometry (GC-MS) [15], Ion exchange chromatography (IEC) [16], high performance liquid chromatography (HPLC) [17,18], and thin layer chromatography (TLC) [19], are based on chromatographic separation, which is a time-consuming process. Moreover, they are usually needed for derivatization processes that can trigger the loss of analytes or make impurities in the detection instruments. Therefore, traditional BA detection approaches suffer from low selectivity, expensive instruments, and the necessity of trained personnel. Moreover, they also suffer from incapability for rapid, real-time, and on-site detection [20]. To address these issues, some new studies have focused on an optical (colorimetric or fluorometric) sensor for sensitive, rapid, simple, and economic BAs’ detection [21,22,23,24]. A major drawback of such optical methods is their poor selectivity, which can be improved by using specific receptors [25,26]. However, the assessment of food freshness needed the detection of multiple BAs simultaneously. In this regard, a colorimetric sensor array can be a fascinating approach for the multi-sensing of Bas [27,28,29]. In a colorimetric sensor array, a distinct pattern is achieved for each analyte by collecting responses from several cross-reactive sensing elements, allowing simultaneous determination of different analytes. Therefore, the fabrication of semi-selective chromophores as cross-reactive sensor elements is the key step in the design of a colorimetric sensor array [30]. Plasmonic nanoparticles have recently gained great attention to use as sensitive and efficient sensor elements due to the interesting analyte-sensitive variations of their surface plasmon resonance (SPR) properties, which produce unique colorimetric patterns [31]. Up to now, using plasmonic NPs as sensor elements has received a considerable amount of attention for the discrimination of different analytes, including pesticides [32,33,34,35,36], biomolecules [37,38,39], and biological drugs [40]. There are also some reports that have applied colorimetric sensor arrays for the discrimination of BAs by using plasmonic NPs [27,29]. However, they usually utilize only AuNPs for providing color patterns for analytes, which requires several sensing elements (some different modifications or morphologies of AuNP) and also may limit the color tonality variation of the array. More recently, there are few reports on designing colorimetric sensor arrays using AuNPs and AgNPs simultaneously for discriminating the diverse analytes such as amyloid peptides [41], proteins [42], organic antifreeze [43], antioxidants [44], and pesticides [34]. Mixing different plasmonic NPs in designing colorimetric sensor array has shown expanded color variation, which enhances the naked eye’s ability to distinguish target analytes. Table S1 represents a number of previous researches that have been able to detect BAs by the colorimetric sensor [20,26,29,45,46,47,48,49,50]. The main drawback of these colorimetric methods, which have been developed based on aggregation of NPs, is that they detect only single Bas [20,26,45,47,49,50,51]. Moreover, there are no reports on using both AuNPs and AgNPs for sensing of BAs. Herein, the different aggregation behaviors of both AuNPs and AgNPs in the presence of target BAs have been exploited to make a unique plasmonic pattern for each BA for their discrimination. Therefore, we have developed a colorimetric sensor array using only two sensor elements for the identification of four BAs. The different compositions of plasmonic NPs (AuNPs or AgNPs) have demonstrated an outstanding color variation in the presence of each target BA, which can discriminate them even in a complex matrix such as a meat product. Moreover, in comparing the figure of merits of this work to the previous colorimetric sensor using plasmonic NPs, the linear range and LOD have been improved [29].

## 2. Experimental

### 2.1. Materials

All materials were analytical reagent grade and were utilized without any purification. Hydrogen tetrachloroaurate (III) trihydrate (HAuCl_4_·3H_2_O) (99.5%), trisodium citrate, silver nitrate, sodium borohydride, spermine, spermidine, histamine, and tryptamine were purchased from Sigma-Aldrich (St. Louis, MO, USA). Milli-Q grade water with a resistivity of 18.2 MΩ was used in all experiments.

### 2.2. Instrumentation

A Perkin Elmer (Lambda25) spectrophotometer was utilized for recording absorbance spectra with a 1.0 cm glass cell. Photographic images were captured by the Samsung A70 smartphone. Controlling of pH was performed with a Denver Instrument Model of 270 pH meters with a Metrohm glass electrode. It is noteworthy that all measurements were obtained at room temperature.

### 2.3. Synthesis of Plasmonic NPs

#### 2.3.1. Synthesis of AuNPs

Citrate-capped AuNPs were synthesized previously by the Turkevich method [52]. Typically, 5.0 mL of trisodium citrate (38.8 mmol L^−1^) were added to the 50 mL of boiled HAuCl_4_ solution (1.0 mmol L^−1^) under vigorous stirring. Then, the solution was mixed for an additional 30 min under reflux condition. The solution was allowed to cool at room temperature and was stored at 4 °C for future use. The prepared AuNPs with the average size of 13 nm showed a plasmonic peak at 520 nm.

#### 2.3.2. Synthesis of AgNPs

Citrate-capped AgNPs were synthesized based on the previous procedure [41]. Briefly, a solution containing 250 μL AgNO_3_ (100 mM), 250 μL trisodium citrate (100 mM), and 100 mL of DI water was prepared. Then, 1 mL of ice-cold NaBH_4_ (5 mM) was added to the prepared solution dropwise under vigorous stirring. The color of the solution was converted to pale yellow and stirred additionally for 30 min. Finally, the synthesized AgNPs were stored at 4 °C in a dark place for 24 h before use. The prepared AgNPs with the average size of 15 nm showed a plasmonic peak at 390 nm.

### 2.4. Design of Sensing Elements

Unmodified AuNPs and AgNPs were utilized as two sensor elements to produce a colorimetric sensor array for the discrimination of BAs. For each of these sensor elements, a 5.0 mL solution containing 350 µL of AuNPs (700 µL of AgNPs) and citrate buffer pH 6.5 with a final concentration of 1.0 mM was prepared. Then, 50 µL of different concentrations of SP, SD, HS, and TP was added to the AuNPs or AgNPs containing solution and it was mixed well. The absorbance spectra were recorded after 10 min.

### 2.5. Real Sample Analysis

To check the practical capability of the developed colorimetric sensor array in a real sample, the target BAs were determined in a meat sample. For this purpose, five pieces of fresh meat (1.0 g) were washed and transferred to five separate 5.0 mL centrifuge tubes. Then, 500 µL of SP, SD, HS, and TP (0.6 mM) was added to four tubes and one tube was considered as a reference sample. The samples were soaked in 5.0 mL of ethanol and shacked by hand for 1 min. In the next step, the samples were centrifuged at 100,000 rpm for three minutes [53]. Finally, 50 µL of each sample was added to the prepared sensor elements and the absorption spectra were recorded after 10 min.

### 2.6. Data Analysis

The statistical data analysis was conducted by Originpro 2018 and MATLAB R2019b. For the qualitative and quantitative detection of the target BAs, principal component analysis-linear discrimination analysis (PCA-LDA) was used to discriminate between the target BAs, and partial least squares regression (PLS-R) was considered to detect the concentration of the target BAs analytes. PLS-toolbox version 7.8 (Eigenvector, Manson, WA, USA) was employed for PLS-R analysis. The prediction accuracy of the models was evaluated using test-set validation. In this regard, the dataset was divided into categories including calibration set (80% of the total data) and test set (20% of total data) using a duplex algorithm. MVC1 toolbox in MATLAB R2019b was exploited for conducting PLS-R analyses and calculating the analytical figures of merit.

## 3. Result and Discussion

We focused on developing colorimetric sensor array for evaluation of meat freshness by monitoring four important BAs. For this purpose, two important plasmonic nanoparticles (AuNPs and AgNPs) were synthesized. The result of the ultraviolet–visible (UV–Vis) spectroscopy with the plasmon absorption band centered at 520 nm (AuNPs) and 390 nm (AgNPs), along with the TEM images of dispersed synthesized AuNPs and AgNPs, confirmed the formation of monodispersed plasmonic gold and silver nanoparticles (Figure S1 in Supplementary Materials). For some of the model BAs, we chose the spermine (SP), spermidine (SD), tryptamine (TP), and histamine (HS), which have different structures and can be considered as chemical indicator of meat freshness. The target BAs can interact differently with the AuNPs or AgNPs due to the dissimilar composition of the NPs and the different chemical structures and functional groups of the BAs. Therefore, this colorimetric sensor array can provide multiplex detection of BAs by using a unique colorimetric pattern for each BA.

### 3.1. Sensing Mechanism of Colorimetric Detection of BAs

**Scheme 1:**
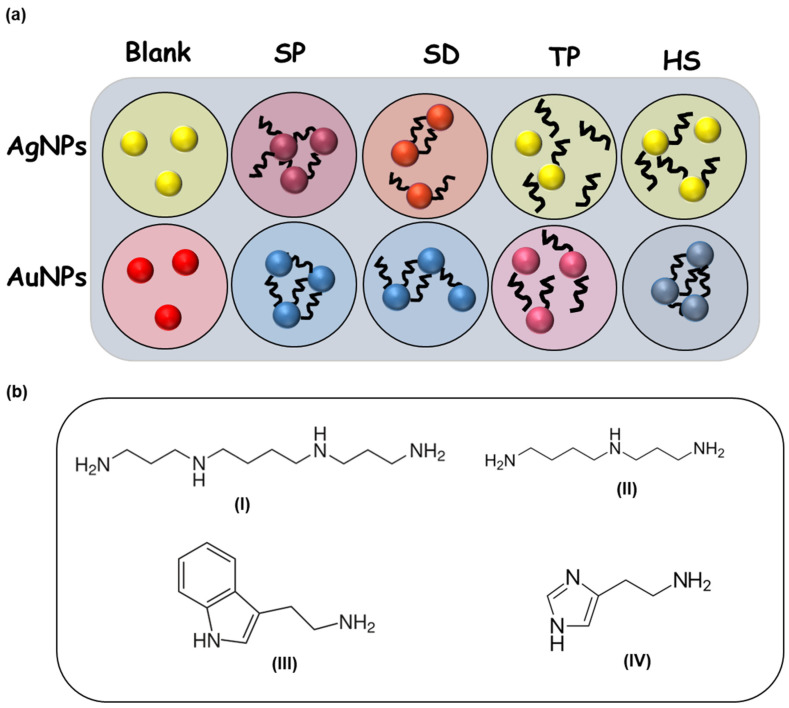
(**a**) Scheme of aggregation AgNPs and AuNPs in the presence of four biogenic amines (**b**) the structures of (I) Spermine (II) Spermidine (III) Tryptamine (IV) Histamine.

As shown in Scheme 1, the target BAs contain primary amines in different structure that can anchor onto the surface of the AuNPs and AgNPs. This binding can be due to the direct interaction between nitrogen and Au (Ag), and it can be considered as well in terms of an electrostatic interaction between the amine groups of BAs and the carboxylic groups of citrates on the NPs, which consequently induce the aggregation of AuNPs and AgNPs [47,50,54].

It is not clear if the interactions of the BAs to the plasmonic NPs are based on electrostatic interaction between amines and carboxylic acid or the chemical binding of amine to the Au or Ag. The electrostatic interaction, including the protonated amino group of BAs and the negatively charged carboxylic acid on the NPs, should be pH dependent. Therefore, the aggregation behaviors of the two sensing elements in the presence of the four target BAs were studied at three pH regimes, namely acidic (4.5), neutral (6.5), and basic (8.5) (Figures S2 and S3). According to their aggregation responses, we found that the AuNPs were aggregated more at acidic pH, which is related to the electrostatic interaction of the protonated BAs at acidic pH and the negative charge of the citrate group (pKa values for citric acids are 3.1, 4.7, and 6.3 [55]) on the AuNPs. Therefore, the electrostatic interaction between amine and carboxylic acid is indeed familiar in the case of the AuNPs. However, the aggregation behaviors of the AgNPs showed that the UV–Vis spectra of the AgNPs did not change in the presence of TP or HS at the all pH regimes, which can confirm that these two BAs did not have an electrostatic interaction. This could be due to the presence of an aromatic ring in their structure, which probably provided a high spatial hindrance to bind to the AgNPs. On the other hand, the aggregation of the AgNPs in the presence of SP and SD with the aliphatic amine groups decreased by increasing the pH values, which can also emphasize the strong interaction of the protonated BAs and the negative charge of the citrate group on the AgNPs.

### 3.2. Optimizing of Experimental Conditions

The primary examination showed that the prepared AuNPs and AgNPs were stable at pH values above 4.0. However, the interaction between the BAs and the prepared AuNPs or AgNPs is pH dependent. According to the aggregation responses of the sensing elements in the presence of the BAs at the three pH regimes (Figures S2 and S3), a neutral environment (pH 6.5) showed a different pattern for each analyte, which can increase the discrimination ability of the developed sensor. However, as illustrated in Figure S2a, the effects of SP, SD, and HS are the same on the AuNPs in acidic pH. Moreover, the aggregation responses of the AgNPs are also the same for SP and SD in acidic pH (Figure S3a). Moreover, the sensitivity of the AgNPs to the target analytes is not enough in alkaline pH (Figure S3c). Therefore, neutral pH was chosen (pH 6.5, citrate buffer (1.0 mM)) as the best pH in developing the colorimetric sensor array. Then, the effect of time was investigated in the absorption spectra of the sensing elements in the presence of the four target analytes. As shown in Figures S4 and S5, the aggregation responses (A_650_/A_520_ in AuNPs and A_500_/A_390_ in AgNPs) were completed within 10 min. A_650_/A_520_ and A_500_/A_390_ were defined as the ratio of the aggregated peak to the plasmon peak of the AuNPs and AgNPs, respectively, which were increased by completing the aggregation of the NPs (Figure S6). Thus, the spectra of both sensing elements were recorded 10 min after the addition of each BA.

### 3.3. Sensor Array Responses

**Figure 1:**
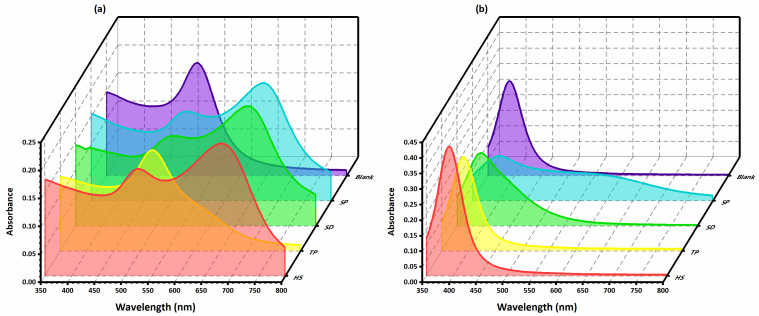
The absorption profile of (**a**) AuNPs and (**b**) AgNPs in the presence of 2.0 µM of BAs (blank (purple), SP (blue) SD (green) TP (yellow) and HS (red)).

**Figure 2:**
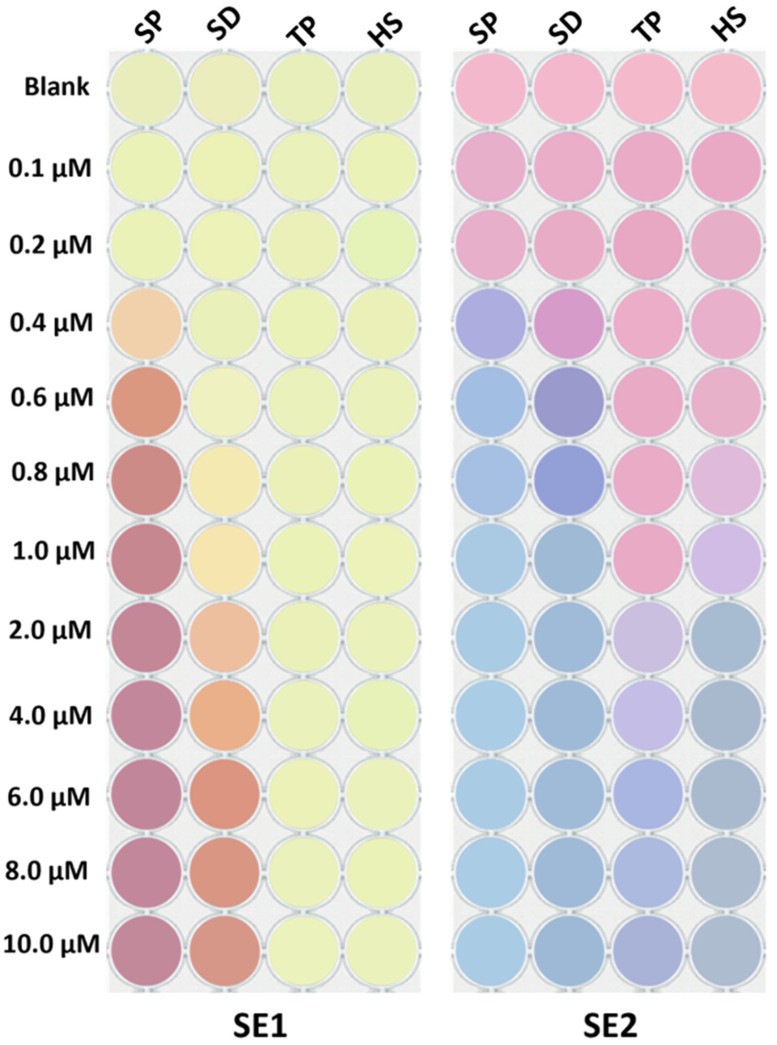
Images taken from the sensing elements upon addition of different concentrations (0.0–10.0 µM) of BAs.

The spectra of the two sensing elements (AuNPs and AgNPs) at optimum conditions (pH 6.5, citrate buffer 1.0 mM, time 10 min) were recorded from 350 to 800 nm in the presence of the wide concentration (0.1 µM–10 µM) of the four BAs (Figures S7 and S8). Representatively, Figure 1 illustrated the changes of the LSPR band of the AuNPs and AgNPs upon the addition of the four BAs at a concentration of 2.0 µM. These observations prove that the type of plasmonic NPs has an important effect on the interaction between the BAs and NPs, which can help the discrimination ability of the developed plasmonic sensor array. For example, while the SP and SD induced a rapid aggregation in the AuNPs, their differences in the aggregation level of the AgNPs cause the discrimination between SP and SD. As compared to SP and SD, two other BAs, namely TP and HS, cause negligible aggregation in the AgNPs. However, the remarkable and different aggregation of the AuNPs causes their distinction. These different behaviors in the aggregation of the AuNPs and AgNPs in the presence of the four BAs can be attributed to the different compositions of plasmonic NPs and also the different structures of the target BAs. Therefore, the different aggregation patterns of the NPs provided the required cross-reactive responses, which can be exploited for the discrimination of the four important BAs. As a result of aggregation, the color of the sensing elements can be changed from red (AuNPs) and yellow (AgNPs) to purple (blue) and orange (red), respectively. Therefore, due to the different spectra patterns, a unique color was also provided for each analyte (Figure 2).

### 3.4. Detection and Discrimination Ability of Plasmonic Sensor Array

**Figure 3:**
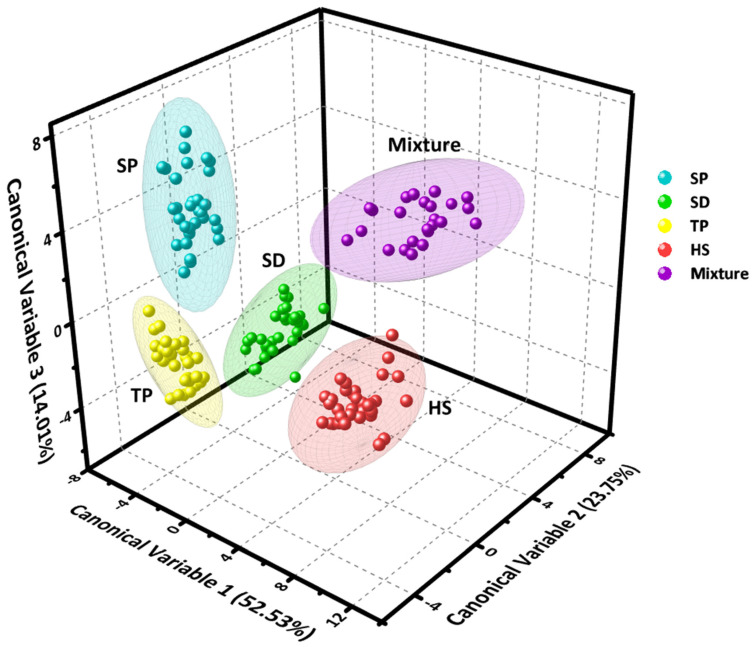
3D Score plot of PCA-LDA for discrimination of four BAs (0.1–10.0 µM) and their mixtures.

The discrimination and regression analysis were applied to a data matrix that is constructed based on the variation of UV–Vis spectra of the AuNPs and AgNPs after the addition of different concentrations of the target analytes. The row and columns of this data matrix are the sample and wavelengths, respectively (156 × 902). It is worth noting that there is a common problem in the LDA model, in which the number of variables should be equal to or less than the number of samples. Therefore, PCA was employed to reduce the high dimensional data of the developed colorimetric sensor (902 variables: 451 wavelengths and 2 NPs) into 20 PCs prior to performing LDA. Thus, PCA-LDA was utilized to analyze the corresponding multidimensional data matrix (156 × 20) to discriminate the four target BAs and as well as their mixtures. Figure 3 presented the 3D score plots of PCA-LDA, which shows the five distinct groups containing the four BAs in a wide concentration range (0.1–10.0 µM) and their mixtures with 100% accuracy (Table S2). Moreover, Figure S9 illustrates the discrimination of the different concentrations of each BA, which showed the excellent potential of the developed colorimetric sensor array not only in the discrimination between the four target BAs and their mixture but also in the discrimination of each concentration of the BAs.

**Figure 4:**
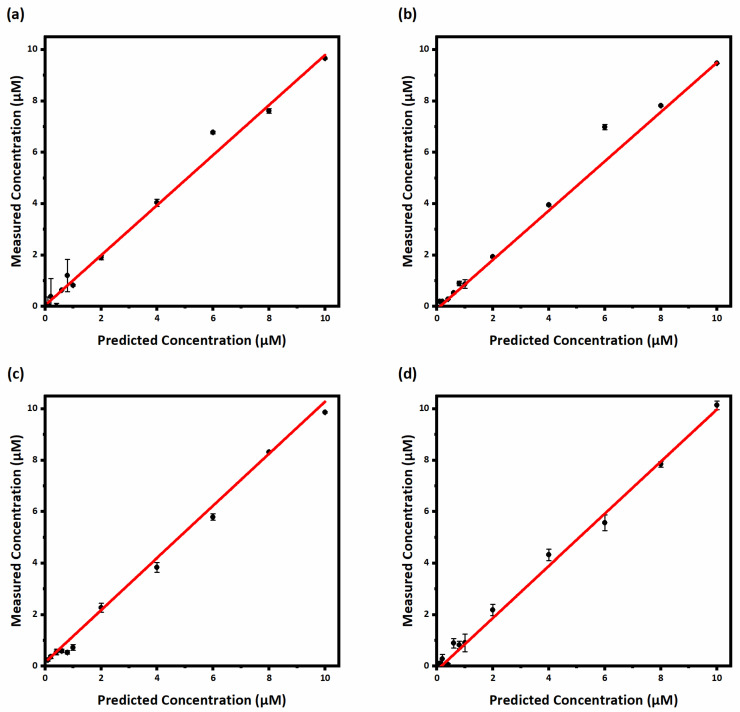
Predicted vs. measured concentration plots by PLS-regression for (**a**) Spermine, (**b**) Spermidine, (**c**) Tryptamine and (**d**) Histamine.

For quantitative analysis, PLS regression (PLS-R) was performed on the data matrix to verify that the developed colorimetric sensor array can predict the concentration of BAs after their recognition. As shown in Figure 4, the predicted concentrations and measured concentrations were successfully correlated with each other, which confirmed the quantitative ability of the colorimetric sensor array. Moreover, the figure of merits that are represented in Table 1 confirmed the satisfaction of the quantitative ability of the colorimetric sensor array. MVC1 toolbox in MATLAB R2019b was exploited for conducting PLS-R analyses and calculating the analytical figures of merit, including correlation coefficient (R^2^), the root-mean-square-error of prediction (RMSEP), sensitivity (SEN), analytical sensitivity (Anal. SEN), limit of detection (LOD), and limit of quantification (LOQ).

### 3.5. Mixture Analysis

**Figure 5:**
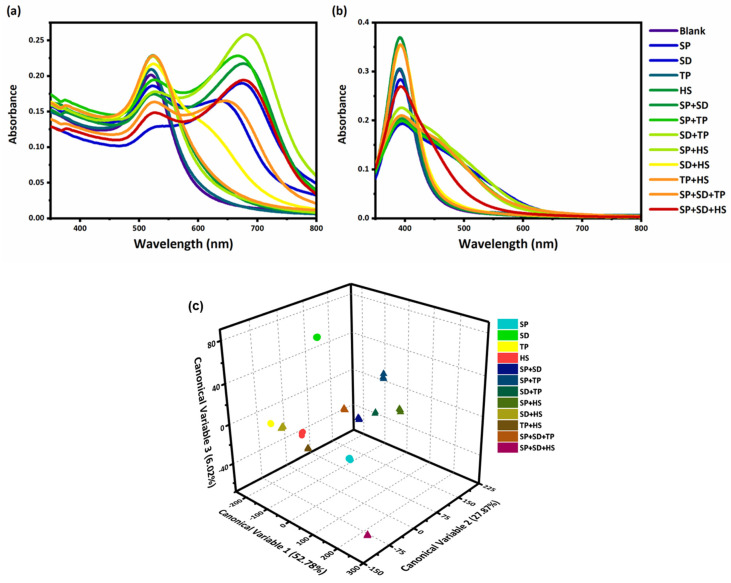
The absorption profile of (**a**) AuNPs and (**b**) AgNPs in the presence pure BAs and the binary and tertiary mixtures of BAs. (**c**) 3D score plots of PCA-LDA for discrimination of different mixtures of BAs. (Circle symbols are related to pure BAs and triangle symbols are related to binary or tertiary mixture samples).

After evaluating the ability of the developed sensor to discriminate the singular analytes in a wide concentration range from their mixtures (Figure 3), the ability of the colorimetric sensor array in the discrimination of each binary or trinary mixtures is one of the significant advantages. Therefore, the absorption spectra of both sensing elements were recorded after the addition of a binary or trinary mixture of BAs (the total concentration of all mixtures was 0.6 µM). As can be seen in Figure 5, their absorption profiles had distinct responses that can be distinguished from their pure samples. The score plot of PCA-LDA in Figure 5c also illustrated that each individual mixture and the pure BAs were discriminated.

### 3.6. Real Sample Analysis

**Figure 6:**
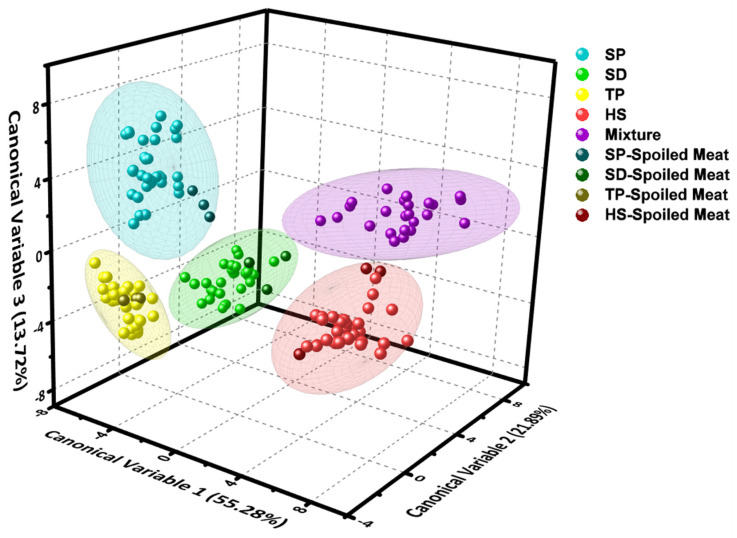
3D score plot of PCA-LDA for identification of BAs in meat sample.

To investigate the feasibility of the developed colorimetric sensor array for the determination of the target BAs in real practical applications, the response absorption of the two sensing elements in the presence of some amino acids and ions, which might be in the meat sample, was evaluated for selectivity purpose (Figure S10). In addition, as shown in Figure S11, these compounds as possible interferences were grouped separately in PCA-LDA. Therefore, they cannot be considered as interferences in the developed colorimetric method. Finally, the ability of the developed nanoplasmonic-based colorimetric sensor array was evaluated in a meat sample as an unknown sample. For this purpose, the AuNPs and AgNPs were exposed to the extracted fresh meat (reference sample) and the BAs to the contaminated meat sample, and their spectra were recorded (Figure S12). It is clearly shown that the fresh meat sample does not provide any changes in the spectra of the sensing elements. Then, the data matrix of these unknown monitored samples was subjected to the PCA-LDA analysis as a test set. The results of PCA-LDA analysis revealed the excellent identification of the BAs in the meat samples (test set) (Figure 6). Moreover, the Mahalanobis distances, a distance of each test set from its centroids of the BA classes in the training, were considered to evaluate the accuracy of the identification of BAs in the meat samples [56]. Table S3 reveals the Mahalanobis distances for each sample and confirms that all unknown samples were assigned to their correct class with an identification accuracy of 100%. Moreover, the recovery values (measured concentration/added concentration × 100) of the developed sensor array were reported as 113.8, 100.8, 88.8, and 110.1 for SP, SD, HS, and TP, respectively, which confirms the potential of this colorimetric sensor in the meat assessment (Table 2).

## 4. Conclusions

In summary, a colorimetric sensor array was proposed for the determination of four important BAs (SP, SD, TP, and HS) as significant chemical indicators of the meat freshness evaluation. In this colorimetric sensor, applying two unmodified plasmonic NPs (AgNPs and AuNPs) improved the expansion of the color variation in the sensing elements’ responses. The results show that the constructed plasmonic sensor array could successfully detect or distinguish the BAs in a wide concentration range. In view of simplicity, sensitivity, and visual readout, our developed sensor can be utilized for the discrimination of the BAs and their mixture by the use of two sensing elements. The results show that this simple colorimetric sensor array can identify the BAs in meat samples, which verifies the ability of this probe in the real samples and the meat freshness analysis. Without using bioenzymes or modifications of the NPs, this simple and cost-effective colorimetric sensor array can be used to verify the quality of meat. Providing portable probes by immobilizing the unmodified NPs on such paper-based substrates can help the researcher for rapid and on-site verification of the quality and health of food products in the near future.

## Figures and Tables

**Table 1:** Analytical figures of merit of PLS regression on BAs.

BAs	LVs	RMSEP	R^2^	SEN	Anal. SEN	LOD min	LOQ min	Linear Range
**SP**	5	0.4200	0.9921	0.0473	16.6448	0.3	0.9	0.1–10.0
**SD**	4	0.3540	0.9944	0.1373	43.1621	0.2	0.6	0.1–10.0
**TP**	5	0.2244	0.9977	0.0822	102.751	0.1	0.4	0.1–10.0
**HS**	8	0.2836	0.9964	0.0140	22.9916	0.2	0.6	0.1–10.0

**Table 2:** Determination of BAs in meat sample.

Sample	Spiked (µmol·L^−1^)	Found (µmol·L^−1^)	Recovery (%)	RSD (n = 3)
**SP**	0.60	0.683	113.9	0.026
**SD**	0.60	0.605	100.8	0.044
**TP**	0.60	0.533	88.8	0.006
**HS**	0.60	0.661	110.1	0.117

## Data Availability

Data Availability Statements are available in section “MDPI Research Data Policies” at https://www.mdpi.com/ethics.

## Supplementary Materials

The following supporting information can be downloaded at: https://www.mdpi.com/article/10.3390/bios13080803/s1. Table S1. Comparison of the proposed methods with a number reported colorimetric methods for BAs detection [26,29,45,46,47,48,49,50,51]. Table S2. Jackknife results of LDA on the training set. Table S3. Identification of unknown BAs in meat sample using PCA-LDA (PCA-LDA was performed on the colorimetric responses of the training set and the meat sample (as the test set). The Mahalanobis distance of the unknown samples from the centroid of the training groups was calculated and used for class identification). Figure S1. The UV-Vis spectra and corresponding TEM images of prepared AuNPs (a,b) and AgNPs (c,d). Figure S2. The absorption spectra of AuNPs in the presence of 2.0 µM of target BAs at three different pH regimes (a) pH 4.5 (b) pH 6.5 (c) pH 8.5. Figure S3. The absorption spectra of AgNPs in the presence of 2.0 µM of target BAs at three different pH regimes (a) pH 4.5 (b) pH 6.5 (c) pH 8.5. Figure S4. The absorption spectra of AuNPs in the presence of target BAs (2.0 µM) after 21 min (time interval between each step is 2 min). Figure S5. The absorption spectra of AgNPs in the presence of target BAs (2.0 µM) after 21 min (time interval between each step is 2 min). Figure S6. The kinetic behavior aggregation response vs time) of (A) AuNPs (SE1) and (B) AgNPs (SE2) in the presence of SP, SD, HS and TP at concentration of 2.0 µM at pH 6.2 (citrate buffer, 1.0 mM). Figure S7. The absorption spectra of AuNPs in the presence of different concentration of (A) Spermine, (B) Spermidine, (C) Tryptamine and (D) Histamine (optimum conditions: citrate buffer (pH 6.5, 1.0 mM), time 10 min). Figure S8. The absorption spectra of AgNPs in the presence of different concentration of (A) Spermine, (B) Spermidine, (C) Tryptamine and (D) Histamine (optimum conditions: citrate buffer (pH 6.5, 1.0 mM), time 10 min). Figure S9. Three-dimensional (3D) score plot of PCA-LDA for discrimination between different concentration (0.1–10.0 µM). Figure S10. The absorption spectra of (a) AuNPs and (b) AgNPs in the presence of meat matrix (blank) and meat contaminated with SP, SD, TP and HS. Figure S11. 3D score plot of PCA-LDA for identification of BAs in the presence possible interferences. Figure S12. The absorption spectra of (a) AuNPs and (b) AgNPs in the presence of meat matrix (blank) and meat contaminated with SP, SD, TP and HS.
