# Supplementary material for: Multiplex Detection of Biogenic Amines for Meat Freshness Monitoring Using Nanoplasmonic Colorimetric Sensor Array
Source: Biosensors (Basel). 2023 Aug 10;13(8):803. doi: 10.3390/bios13080803 (PMC10452313; doi:10.3390/bios13080803)
Supplement: Supplementary file 1 [file biosensors-13-00803-s001.zip › biosensors-2483420-supplementary.pdf]

Supporting Information

Multiplex Detection of Biogenic Amines for Meat Freshness Monitoring Using Nanoplasmonic Colorimetric Sensor Array

Samira Abbasi-Moayed[a], Afsaneh Orouji[b] and M. Reza Hormozi-Nezhad[b,c*]*

[a] Department of analytical chemistry, Faculty of chemistry, Kharazmi University, 15719-14911, Tehran, Iran

[b] Department of Chemistry, Sharif University of Technology, Tehran, 111559516, Iran

[c] Institute for Nanoscience and Nanotechnology, Sharif University of Technology, Tehran, 11155-9516, Iran

*Corresponding authors:

- M. Reza Hormozi-Nezhad, email: hormozi@sharif.edu

ORCID iD: 0000-0002-7472-1850

-Samira Abbasi-Moayed, email: s.abbasimoayed@khu.ac.ir

Table of contents

Content	Page
**Figure S1.** The UV-Vis spectra and corresponding TEM images of prepared AuNPs (a, b) and AgNPs (c, d)	**S3**
**Figure S2.** The absorption spectra of AuNPs in the presence of 2.0 μM of target BAs at three different pH regimes (a) pH 4.5 (b) pH 6.5 (c) pH 8.5	**S4**
**Figure S3.** The absorption spectra of AgNPs in the presence of 2.0 μM of target BAs at three different pH regimes (a) pH 4.5 (b) pH 6.5 (c) pH 8.5	**S5**
**Figure S4.** The absorption spectra of AuNPs in the presence of target BAs (2.0 μM) after 21 minutes (time interval between each step is 2 minute)	**S6**
**Figure S5.** The absorption spectra of AgNPs in the presence of target BAs (2.0 μM) after 21 minutes (time interval between each step is 2 minute)	**S7**
**Figure S6.** The kinetic behavior aggregation response vs time) of (A) AuNPs (SE1) and (B) AgNPs (SE2) in the presence of SP, SD, HS and TP at concentration of 2.0 μM at pH 6.2 (citrate buffer, 1.0 mM)	**S8**
**Figure S7.** The absorption spectra of AuNPs in the presence of different concentration of (A) Spermine, (B) Spermidine, (C) Tryptamine and (D) Histamine (optimum conditions: citrate buffer (pH 6.5, 1.0 mM), time 10 min)	**S9**
**Figure S8.** The absorption spectra of AgNPs in the presence of different concentration of (A) Spermine, (B) Spermidine, (C) Tryptamine and (D) Histamine (optimum conditions: citrate buffer (pH 6.5, 1.0 mM), time 10 min)	**S10**
**Table S2.** Jackknife results of LDA on the training set	**S11**
**Figure S9.** Three-dimensional (3D) score plot of PCA-LDA for discrimination between different concentration (0.1-10.0 μM)	**S12**
**Figure S10.** The absorption spectra of (a) AuNPs and (b) AgNPs in the presence of meat matrix (blank) and meat contaminated with SP, SD, TP and HS.	**S13**

Table S3. Identification of unknown BAs in meat sample using PCA-LDA (PCA-LDA was performed on the colorimetric responses of the training set and the meat sample (as the test set). The Mahalanobis distance of the unknown samples from the centroid of the training groups was calculated and used for class identification). **S14**

Table S1. Comparison of the proposed methods with a number reported colorimetric methods for BAs detection.

**Analyte**	**Probe**	**Mechanism**	**Linear range (μM)**	**LOD (μM)**	**Real Sample**	**Reference**
Histamine	AuNPs	Aggregation	0.1-2.1	0.04	Salmon fish	[1]
Histamine	AuNPs	Aggregation	2.0-16.0	0.6	Chicken	[2]
Histamine	AuNPs	Aggregation	0.2-0.4	0.2	Wine	[3]
Histamine	AuNPs	Growth	9.0-90.0	25.2	Shrimp	[4]
Spermine	AuNPs	Aggregation	0.1-2.0	0.01	Beef	[5]
Spermine	hydroxyl pillar[5]arene/AuNPs	Aggregation	0.1-4.0	0.03	-	[6]
Spermine	AuNPs	Aggregation	0.03-0.07	0.05	Human Urine	[7]
Spermine	6-azo-2-thiothymine/AgNPs	Aggregation	2.5-20	0.23	Urine	[8]
**Spermine**<br>**Tryptamine**<br>**Ethylenediamine**<br>**Tyramine**<br>**Spermidine**<br>Histamine	AuNRs and AuNPs	Growth	20-800<br>40-800<br>60-800<br>80-800<br>10-800<br>40-800	4.7<br>8.6<br>10.1<br>27.3<br>2.5<br>14.3	Salmon fish	[9]
Spermine<br>**Spermidine**<br>**Tryptamine**<br>Histamine	AuNPs and AgNPs	Aggregation	0.1-10	0.3<br>0.2<br>0.1<br>0.2	Meat	This work

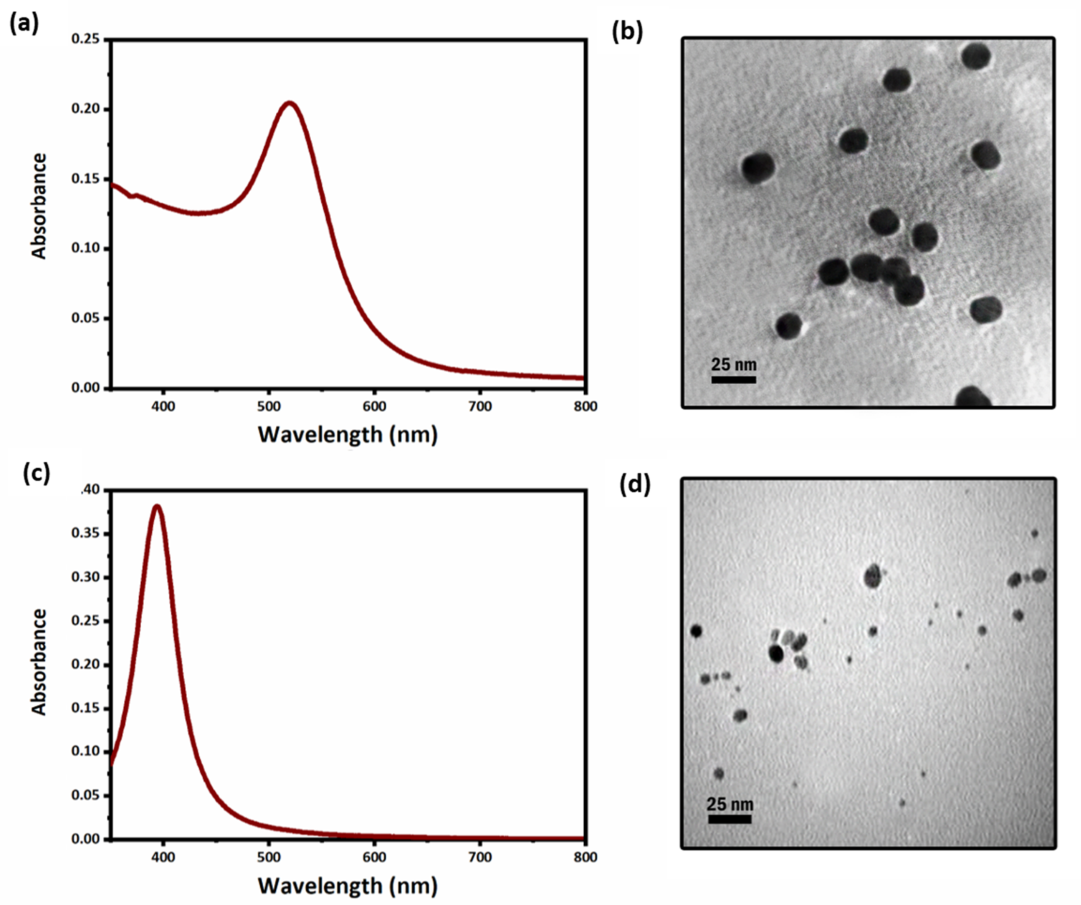

Figure S1. The UV-Vis spectra and corresponding TEM images of prepared AuNPs (a, b) and AgNPs (c, d).

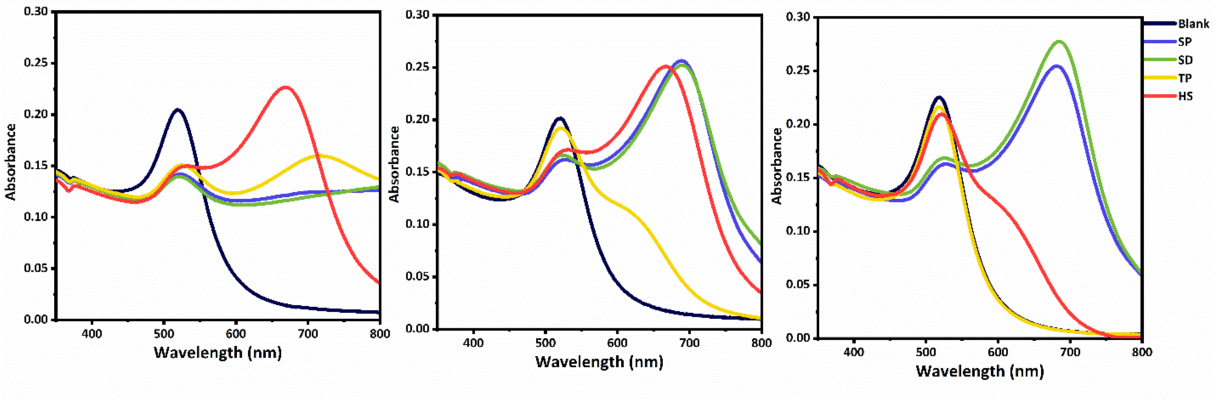

Figure S2. The absorption spectra of AuNPs in the presence of 2.0 μM of target BAs at three different pH regimes (a) pH 4.5 (b) pH 6.5 (c) pH 8.5.

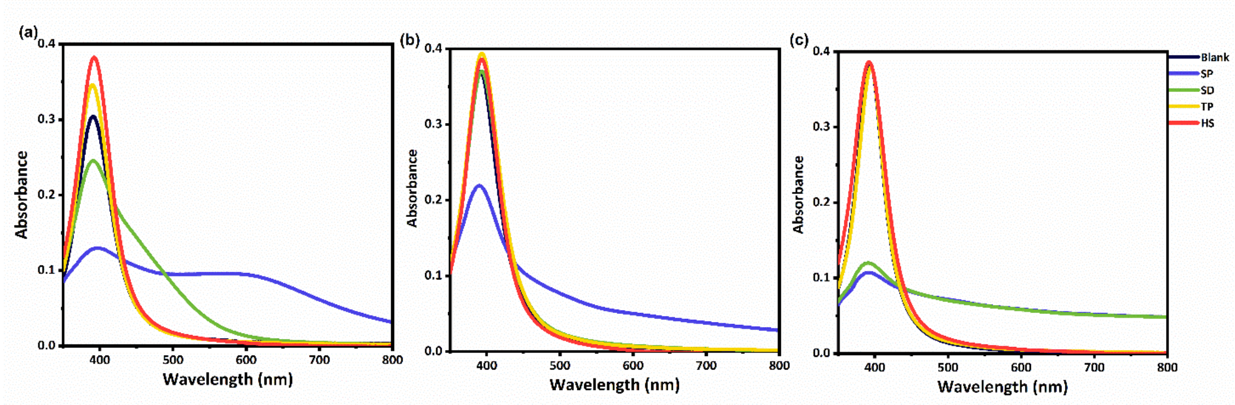

Figure S3. The absorption spectra of AgNPs in the presence of 2.0 μM of target BAs at three different pH regimes (a) pH 4.5 (b) pH 6.5 (c) pH 8.5.

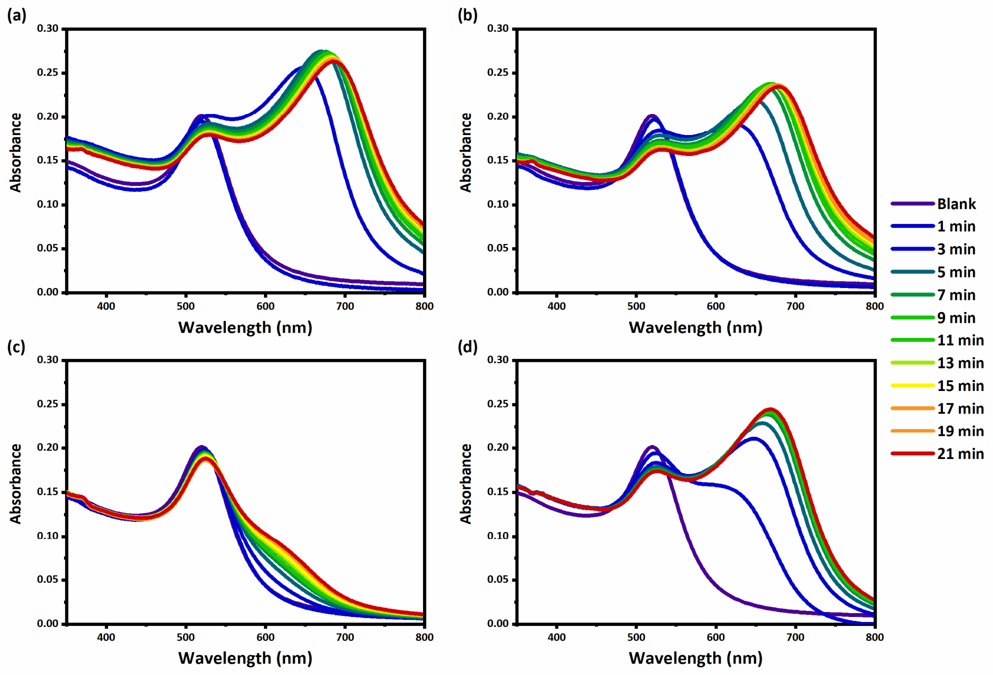

Figure S4. The absorption spectra of AuNPs in the presence of (a) SP (b) SD (c) TP and (d) HS at the concentration of 2.0 μM after 21 minutes (time interval between each step is 2 minute).

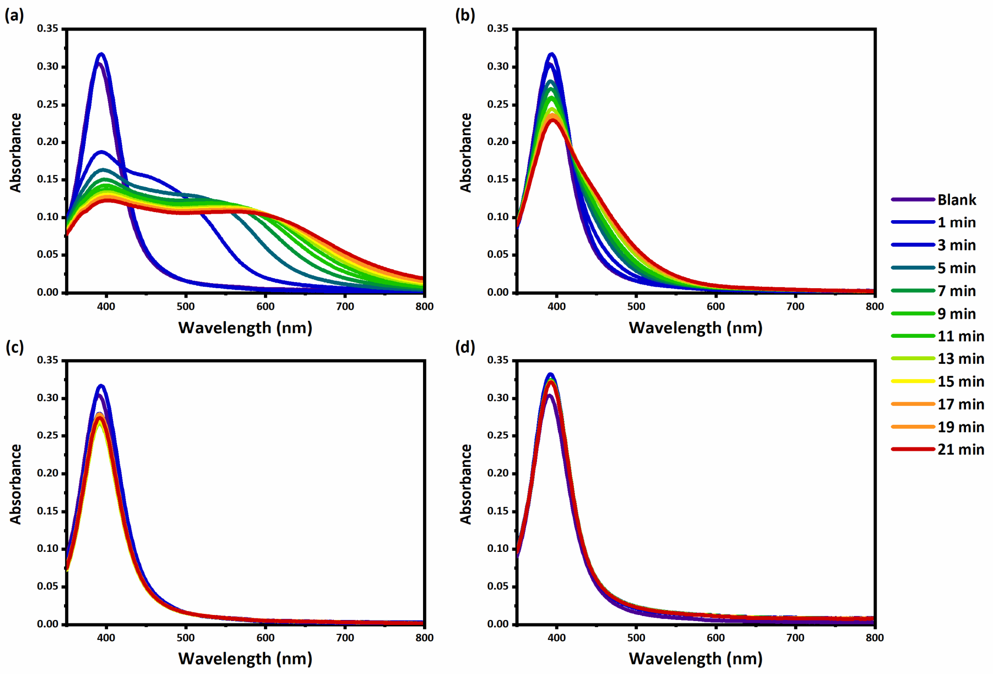

Figure S5. The absorption spectra of AgNPs in the presence of (a) SP (b) SD (c) TP and (d) HS at the concentration of 2.0 μM after 21 minutes (time interval between each step is 2 minute).

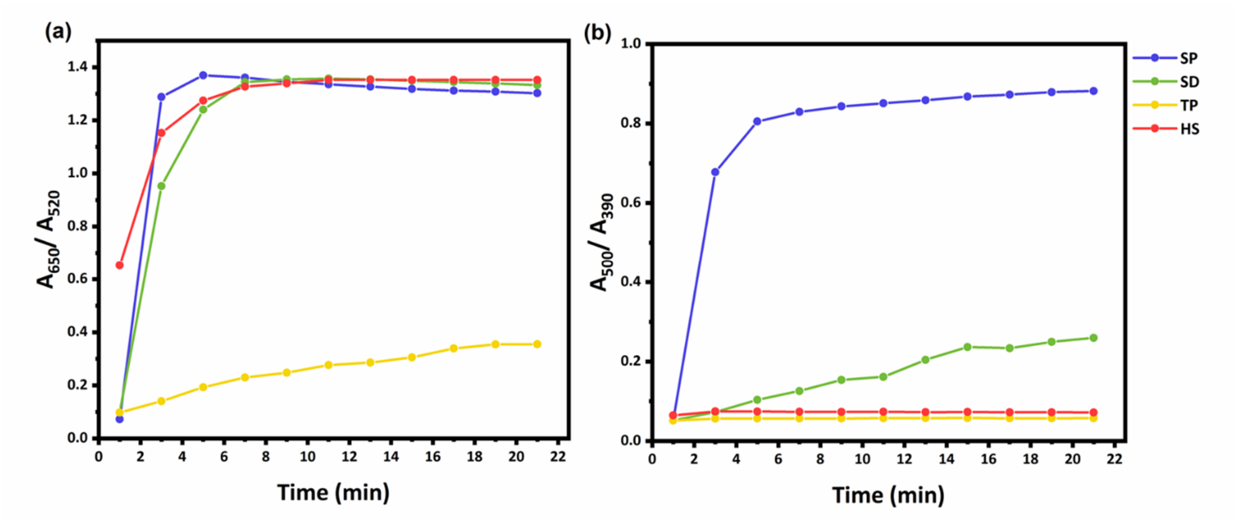

Figure S6. The kinetic behavior aggregation response vs time) of (A) AuNPs (SE1) and (B) AgNPs (SE2) in the presence of SP, SD, HS and TP at concentration of 2.0 μM at pH 6.2 (citrate buffer, 1.0 mM).

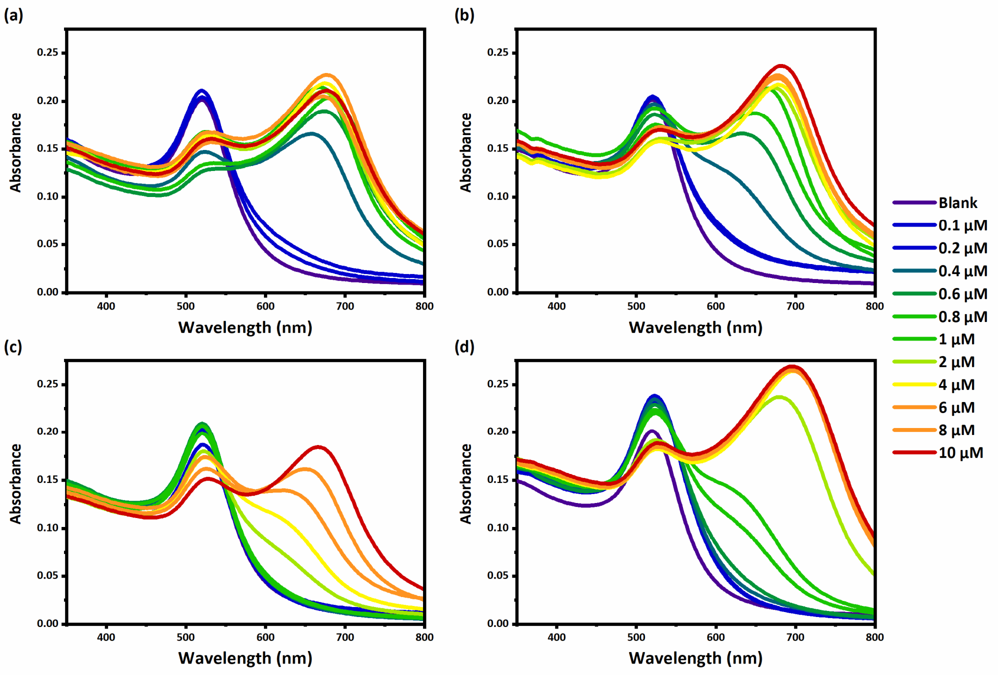

Figure S7. The absorption spectra of AuNPs in the presence of different concentration of (A) Spermine, (B) Spermidine, (C) Tryptamine and (D) Histamine (optimum conditions: citrate buffer (pH 6.5, 1.0 mM), time 10 min).

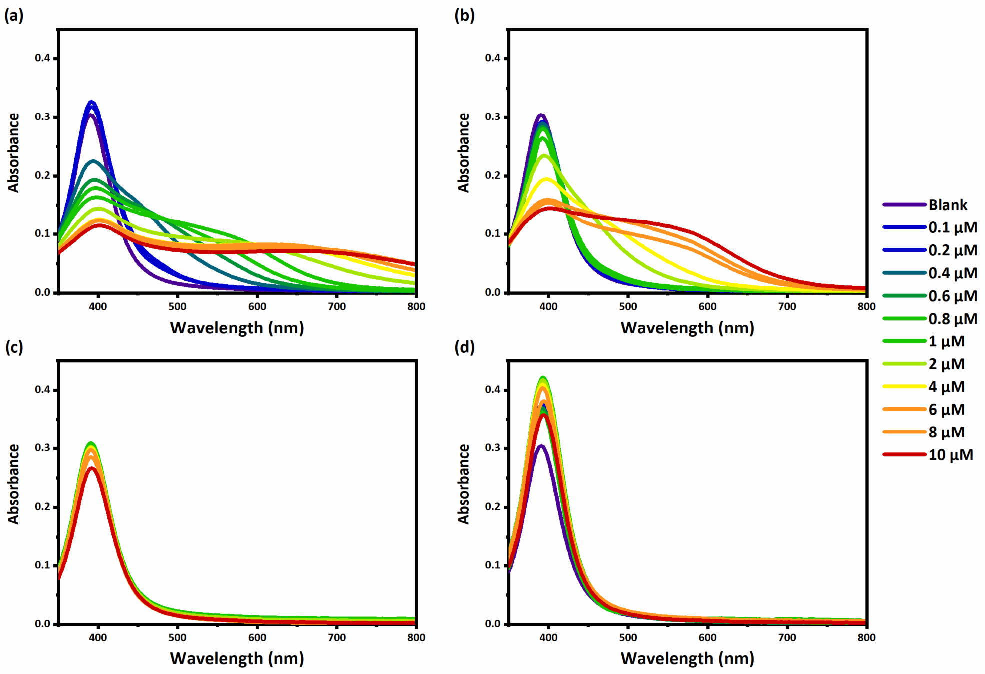

Figure S8. The absorption spectra of AgNPs in the presence of different concentration of (A) Spermine, (B) Spermidine, (C) Tryptamine and (D) Histamine (optimum conditions: citrate buffer (pH 6.5, 1.0 mM), time 10 min).

Table S2. Jackknife results of LDA on the training set.

		Predicted Class					
		SP	SD	TP	HS	Mixture	Total
Actual Class	SP	33	0	0	0	0	33
		100.0%	0.00%	0.00%	0.00%	0.00%	100.0%
	SD	0	33	0	0	0	33
		0.00%	100.0%	0.00%	0.00%	0.00%	100.0%
	TP	0	0	33	0	0	33
		0.00%	0.00%	100.0%	0.00%	0.00%	100.0%
	HS	0	0	0	33	0	33
		0.00%	0.00%	0.00%	100.0%	0.00%	100.0%
	Mixture	0	0	0	0	24	24
		0.00%	0.00%	0.00%	0.00%	100.0%	100.0%
	Total	33	33	33	33	24	156
		21.15%	21.15%	21.15%	21.15%	15.4%	100.0%

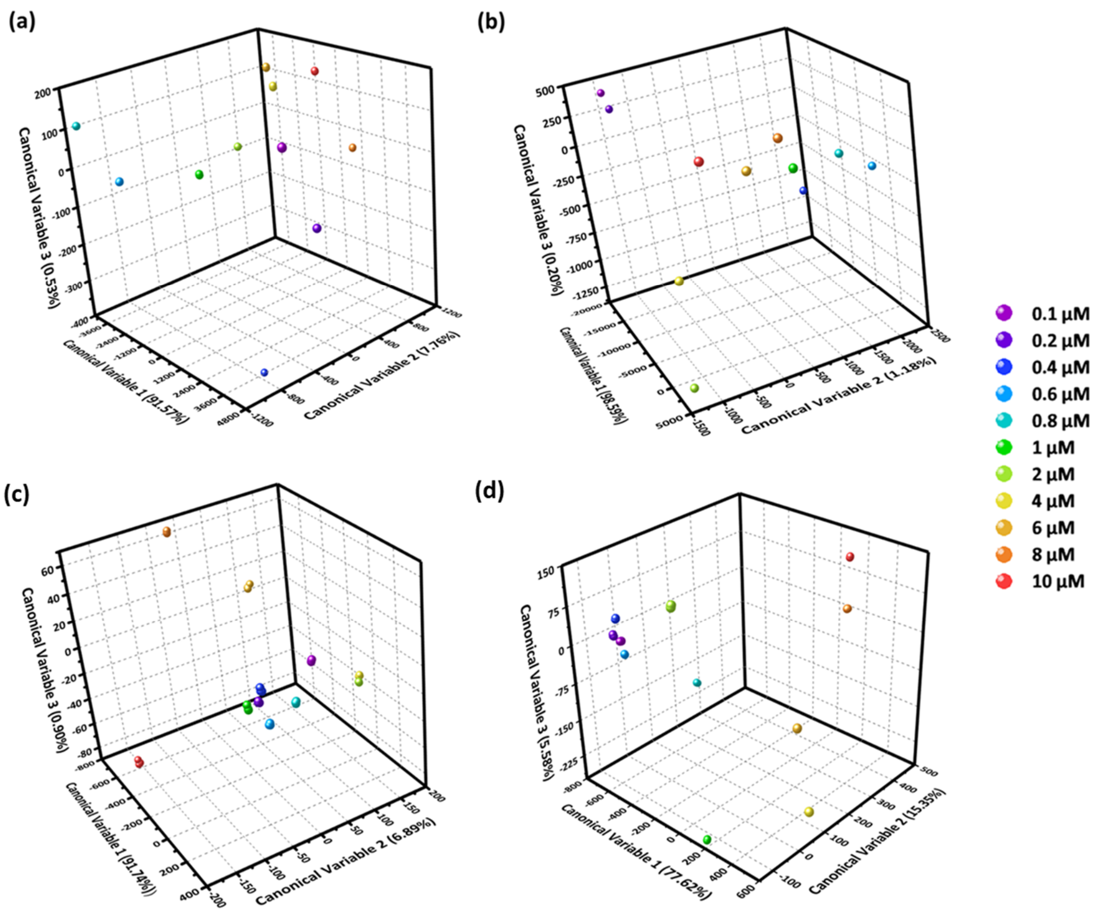

Figure S9. Three-dimensional (3D) score plot of PCA-LDA for discrimination between different concentration (0.1-10.0 μM).

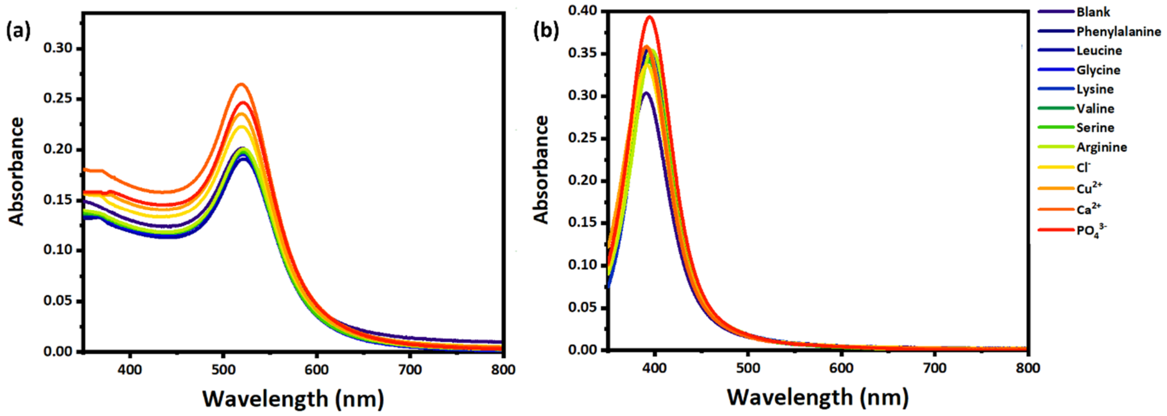

Figure S10. The absorption spectra of (a) AuNPs and (b) AgNPs in the presence of different amino acid and ions.

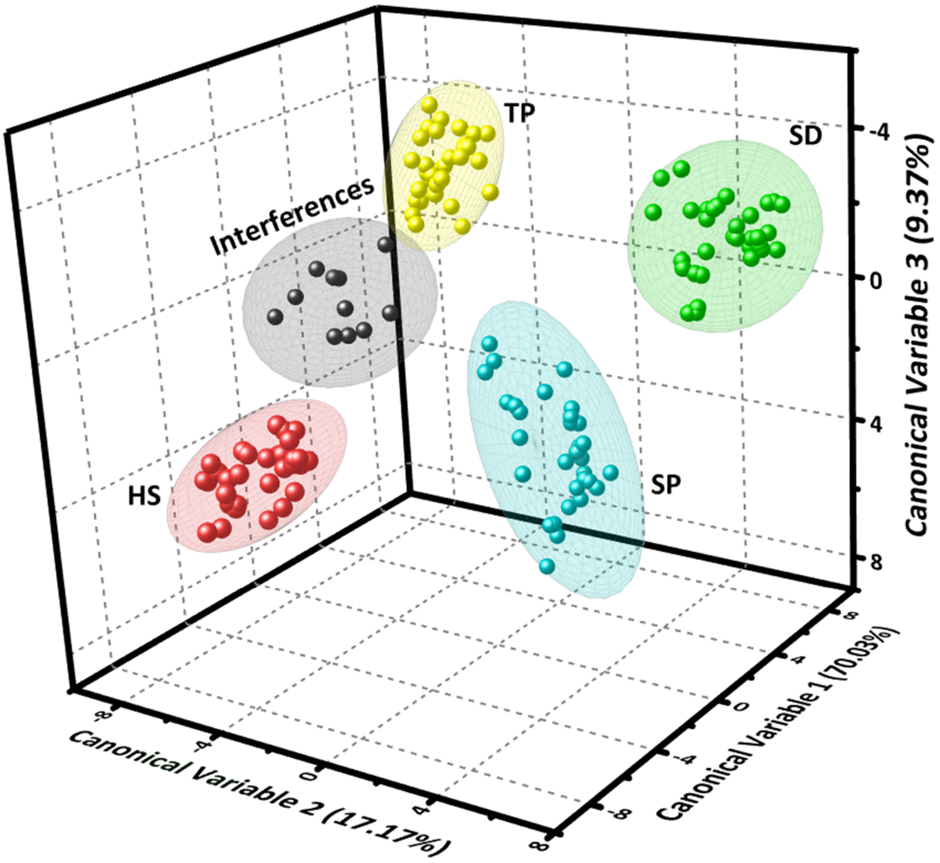

Figure S11.3D score plot of PCA-LDA for identification of BAs in the presence possible interferences.

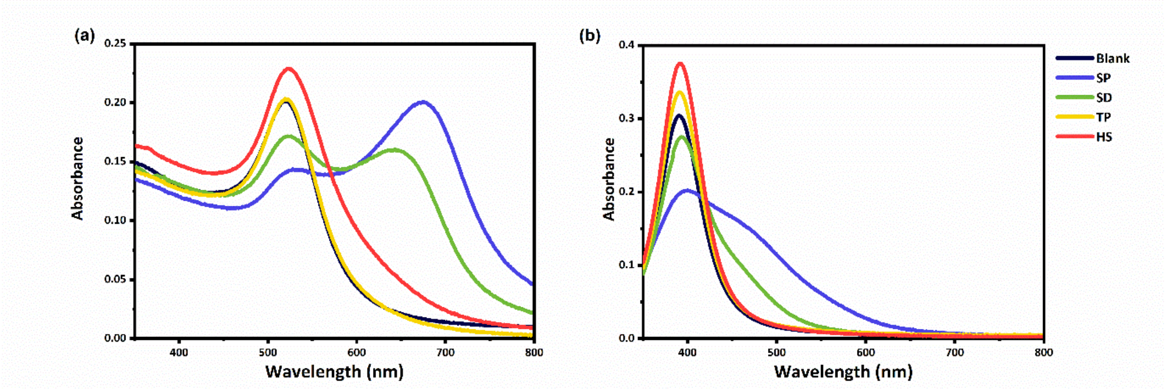

Figure S12. The absorption spectra of (a) AuNPs and (b) AgNPs in the presence of meat matrix (blank) and meat contaminated with SP, SD, TP and HS.

Table S3. Identification of unknown BAs in meat sample using PCA-LDA (PCA-LDA was performed on the colorimetric responses of the training set and the meat sample (as the test set). The Mahalanobis distance of the unknown samples from the centroid of the training groups was calculated and used for class identification).

Sample	Mahalanobis distance-square					Identified	Accuracy of identification
	SP	SD	TP	HS	Mixture		
1	47.50067	63.23994	118.5535	208.15811	172.89643	SP	Yes
2	52.40421	63.31959	105.6814	187.19098	157.18552	SP	Yes
3	45.9406	72.51988	135.697	234.86279	197.93355	SP	Yes
4	126.04729	78.52219	158.53261	162.00655	139.82409	SD	Yes
5	152.15811	93.48668	157.94727	168.32184	172.59615	SD	Yes
6	125.36516	108.26037	134.8782	156.70104	159.01085	SD	Yes
7	75.52525	104.73279	13.03061	120.89905	157.65675	TP	Yes
8	83.35536	111.94516	6.92949	149.2826	168.21905	TP	Yes
9	79.83879	108.52752	8.73245	130.72524	156.22293	TP	Yes
10	187.38392	167.27084	169.54067	29.76728	61.7933	HS	Yes
11	181.86381	172.87234	166.20817	32.67613	73.01	HS	Yes
12	194.82008	169.52054	110.0658	16.77327	113.76953	HS	Yes

References

1. Rawat, K.A., et al., *Microwave assisted synthesis of tyrosine protected gold nanoparticles for dual (colorimetric and fluorimetric) detection of spermine and spermidine in biological samples.* Biosensors and Bioelectronics, 2017. **88**: p. 71-77.
2. El-Nour, K., et al., *Gold nanoparticles as a direct and rapid sensor for sensitive analytical detection of biogenic amines.* Nanoscale research letters, 2017. **12**: p. 1-11.
3. Lapenna, A., et al., *"Naked" gold nanoparticles as colorimetric reporters for biogenic amine detection.* Colloids and Surfaces A: Physicochemical and Engineering Aspects, 2020. **600**: p. 124903.
4. Li, H., et al., *Colorimetric detection of food freshness based on amine-responsive dopamine polymerization on gold nanoparticles.* Talanta, 2021. **234**: p. 122706.
5. Wang, J., et al., *Selective colorimetric analysis of spermine based on the cross-linking aggregation of gold nanoparticles chain assembly.* Talanta, 2017. **167**: p. 193-200.
6. Tan, X., et al., *Colorimetric sensing towards spermine based on supramolecular pillar [5] arene reduced and stabilized gold nanoparticles.* Spectrochimica Acta Part A: Molecular and Biomolecular Spectroscopy, 2019. **221**: p. 117176.

7. Jornet-Martínez, N., et al., *Sensitive and selective plasmonic assay for spermine as biomarker in human urine.* Analytical chemistry, 2014. **86**(3): p. 1347-1351.
8. Kailasa, S.K., et al., *Independent spectral characteristics of functionalized silver nanoparticles for colorimetric assay of arginine and spermine in biofluids.* New Journal of Chemistry, 2019. **43**(43): p. 17069-17077.
9. Orouji, A., et al., *Providing multicolor plasmonic patterns with Au@ Ag core–shell nanostructures for visual discrimination of biogenic amines.* ACS Applied Materials & Interfaces, 2021. **13**(17): p. 20865-20874.
